# *EMD* missense variant causes X-linked isolated dilated cardiomyopathy with myocardial emerin deficiency

**DOI:** 10.1038/s41431-025-01827-8

**Published:** 2025-03-10

**Authors:** Linda Bulmer, Charlotta Ljungman, Johan Hallin, Pia Dahlberg, Christian L. Polte, Carola Hedberg-Oldfors, Anders Oldfors, Anders Gummesson

**Affiliations:** 1 Department of Clinical Genetics and Genomics, Sahlgrenska University Hospital, Gothenburg, Sweden; 2 Department of Molecular and Clinical Medicine, Institute of Medicine, Sahlgrenska Academy, University of Gothenburg, Gothenburg, Sweden; 3 Department of Cardiology, Sahlgrenska University Hospital, Gothenburg, Sweden; 4 Departments of Clinical Physiology and Radiology, Sahlgrenska University Hospital, Gothenburg, Sweden; 5 Department of Laboratory Medicine, Institute of Biomedicine, Sahlgrenska Academy, University of Gothenburg, Gothenburg, Sweden

**Keywords:** Cardiomyopathies, Ventricular tachycardia, Heart failure, Neuromuscular disease, Disease genetics

## Abstract

Pathogenic variants in the *EMD* gene cause X-linked Emery–Dreifuss muscular dystrophy type 1 (EDMD1), typically presenting with joint contractures and skeletal muscle atrophy, followed by atrial arrhythmias, cardiac conduction defects, and atrial dilatation. Although an association with isolated dilated cardiomyopathy (DCM) has been suggested, evidence is currently insufficient to verify the gene-disease association. We investigated the causality of a missense variant, c.23C>G, p.Ser8Trp, in *EMD* in a large family with a history of DCM and suspected sudden cardiac death (SCD) in males. DCM was diagnosed in six hemizygous males aged 36–50 and detailed phenotyping identified end-stage heart failure, cardiac conduction defects, and ventricular arrhythmias as prominent features. Cardiac magnetic resonance imaging showed late gadolinium enhancement with mixed ischemic and non-ischemic patterns. Muscular dystrophy was absent in all six males, of whom five underwent neuromuscular examination including serum-creatine kinase measurement. Immunohistochemical analysis showed greatly reduced levels of emerin in both cardiac and skeletal muscle samples. The *EMD* variant c.23C>G co-segregated with DCM, with an estimated LOD score of 3.9 and full-likelihood Bayes factor of >2500:1 in favor of causality. Among the 17 heterozygous females, ages 20–87, one developed DCM at age 72. We concluded that the *EMD* c.23C>G missense variant is associated with DCM in the absence of muscular dystrophy, thereby providing new evidence of isolated DCM as a distinct cardiac *EMD*-phenotype, separate from EDMD1. The phenotypic similarities with *LMNA*-DCM, with a high risk of cardiac conduction defects and ventricular arrhythmias, might warrant early interventions to prevent SCD.

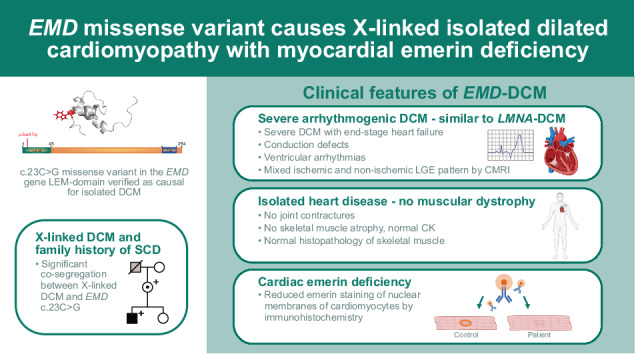

## Introduction

Dilated cardiomyopathy (DCM) is defined by left or biventricular dilatation and systolic dysfunction not explained by abnormal loading conditions or coronary artery disease [1]. In the 20–35% of idiopathic DCM cases estimated to be familial [2], identification of the underlying genetic cause is necessary to enable individualized clinical management and pre-symptomatic testing of at-risk family members. However, 60–85% of familial DCM cases remain unexplained after conventional genetic screening [3]. Furthermore, among genes reported to associated with monogenic DCM, relatively few are supported by robust evidence of co-segregation or impact on protein function [4].

The *EMD* gene, encoding the globally expressed nuclear envelope protein emerin [5], has so far only been established as causative for DCM as a feature of Emery–Dreifuss muscular dystrophy type 1 (EDMD1) (MIM # 310300) [6]. EDMD1 is a rare X-linked disorder characterized by childhood onset of joint contractures and progressive muscular atrophy followed by cardiac disease from adolescence [7]. The cardiac phenotype of EDMD1 is typically dominated by atrial arrhythmias, cardiac conduction defects, and atrial dilatation [8–10]. Mild left ventricular dilatation and systolic dysfunction are common [11, 12] and new evidence suggests that ventricular involvement might be more severe than previously reported [13]. However, DCM is rarely reported in EDMD1 [14].

The degree of neuromuscular involvement in EDMD1 shows both inter- and intrafamilial variability [15] and there are reports of cardiac disease being the predominant manifestation in males harboring *EMD* variants [16–20]. However, studies linking isolated DCM to *EMD* are scarce [21, 22] and no *EMD* variant has yet been shown to be causative for isolated DCM by combined evidence of co-segregation and emerin deficiency. Furthermore, there are data that contradict that *EMD* plays a role in isolated DCM, including a large cohort study which concluded that the prevalence of rare *EMD* variants did not differ between DCM patients and controls [23]. Consequently, *EMD* is not listed among the known genes associated with non-syndromic familial DCM in current guidelines on genetic screening in DCM [24].

In this study, we applied segregation analysis and emerin immunostaining on myocardial tissue to verify the causality of a missense variant in the *EMD* gene to X-linked DCM without muscular dystrophy in a large family, thereby providing new evidence linking the *EMD* gene to isolated DCM.

## Methods

### Study participants

The Swedish family was investigated at the Cardiogenetic clinic at Sahlgrenska University Hospital between 2015 and 2024, and the study included adult family members. Written informed consent was obtained from all participants prior to inclusion in the study. Medical records from deceased family members were evaluated after obtaining consent from close relatives.

### Phenotype characterization

Results of the clinical investigations were obtained from medical records. Cardiac magnetic resonance imaging (CMRI) exams were reanalyzed based on current recommendations using IntelliSpace (Philips Healthcare, Best, The Netherlands). DCM was diagnosed by the presence of left ventricular dilatation and systolic dysfunction in the absence of hypertension, valvular disease, or coronary artery disease sufficient to cause global systolic impairment, or congenital heart disease [1]. When available, left ventricular dilatation was diagnosed according to indexed left ventricular end-diastolic volume by CMRI. For cardiac ultrasound, a left ventricular end-diastolic diameter above 58 mm for males and above 52 mm for females was considered diagnostic. Systolic dysfunction was diagnosed as ejection fraction below 50%. Other causes of DCM were excluded according to clinical routine, as presented in Supplementary Table 1.

All males with DCM, except the deceased, were examined by a specialist in neurology at the Centre for Neuromuscular Disorders at Sahlgrenska University Hospital. Evaluations included physical examination (including assessment of joint contractures and skeletal muscle atrophy), neurological status, and serum-creatine kinase measurement. Histopathological examination following deltoid muscle biopsy was performed on two males.

### Genetic analyses

Genetic screening was performed by targeted sequencing of exon and exon-intron boundaries using a commercial gene panel for DCM (Blueprint Genetics, Helsinki, Finland). Gene lists are available in Supplementary Table 2. Cascade screening by targeted testing for the detected *EMD* variant c. 23C>G, p.Ser8Trp (NM_000117.3) was conducted for relatives at risk of inheriting the variant, either via NGS (Blueprint Genetics) or Sanger sequencing. Sanger sequencing was performed using BigDye Terminator v3.1 cycle sequencing kit (ThermoFisher Scientific, Waltham, MA, USA) on an ABI 3500xl Genetic Analyzer (ThermoFisher Scientific). In male participants unavailable for testing, the presence of the *EMD* variant was determined indirectly by testing their daughters, if possible.

Whole genome sequencing was performed to investigate the occurrence of other rare variants linked to the *EMD* locus. DNA extracted from blood was prepped using TruSeq DNA PCR-free (Illumina, San Diego, CA, USA) and sequenced using NovaSeq 6000 (Illumina) to a genome-wide average coverage of 44×. Bioinformatic analysis was performed at SciLife Clinical Genomics (Gothenburg, Sweden) with variant calling for SNPs and indels performed using DNA-scope (Sentieon, San Jose, CA, USA) and copy number analysis using CANVAS (Illumina). All genes within 1 Mb upstream and downstream of *EMD* were manually curated and six were identified as potentially related to DCM (*FLNA, TAFAXZZIN, CLIC2, BGN, SLC6A8*, and *MECP2*). All variants in *EMD* and the listed genes ±1 Kb were extracted (84 variants total). Variants with an allele frequency above 1% in GnomAD v2.1.1 [25] were filtered out leaving only two variants: the *EMD* variant c.23C>G and an intronic 1 bp deletion in the *FLNA* gene, with no predicted effect on splicing (SpliceAI) [26].

### Segregation analysis

The association between the *EMD* c.23C>G variant and DCM was investigated by segregation analysis in male family members with known genotype and phenotype. Their phenotypes were denoted as affected, unaffected, or unknown based on the phenotype characterization. Those fulfilling the diagnostic criteria for DCM were classified as affected regardless of age. For those not diagnosed with DCM, the phenotype was denoted as unaffected only if they were 60 years or older. This age threshold was based on family history and previous reports on the age of onset of cardiac disease in EDMD1 [7, 16–19, 21, 27, 28]. Younger males (<60 years) without DCM were denoted as unknown phenotypes due to the uncertainty regarding DCM development later in life. Pedigrees used for the segregation analyses are shown in Supplementary Figs. 1 and 2.

The estimated LOD score was calculated using the following formula, with *N* denoting the number of informative meioses/segregations in the pedigree:

$${{{\rm{Z}}}}({{{\rm{LOD\; score}}}})=\log 10\frac{1}{{0,5}^{N}}$$

*Z* values > 3.0 were considered evidence of linkage between the *EMD* locus and DCM, and values ≤ 2.0 were considered evidence against linkage.

Full-likelihood Bayes factor (FLB) was calculated according to the method presented by Thompson et al. [29] using the segregatr package in R [30]. Calculations were performed for the whole pedigree using penetrance 100%, phenocopy rate 1/250 [31], and allele frequency 0.000001. Recognizing the uncertainty of the chosen parameters, the analysis was recalculated systematically using values ranging from 90 to 100% for penetrance, 1/100 to 1/2000 for phenocopy rate, and 0.0000005 to 0.00001 for allele frequency.

### Immunohistochemical analyses on cardiac and skeletal muscle specimens

Cardiac and skeletal muscle specimens were collected in a clinical setting. The explanted hearts were fixed in 4% paraformaldehyde and selected parts were embedded in paraffin. The muscle biopsies were snap-frozen in liquid propane chilled with liquid nitrogen. Sections were cut in a cryostat for histological and histochemical analyses applying standard techniques [32]. Emerin immunohistochemistry was conducted using the NCL-Emerin mouse monoclonal antibody (Leica Biosystems, Newcastle, UK) at 1:500 dilution. The immunostaining was performed using a Dako Autostainer with EnVision FLEX (Agilent, Santa Clara, CA, USA) visualization.

### Western blot analysis on skeletal muscle specimen

Western blot analysis of emerin was performed on protein extracted from sections of fresh-frozen skeletal muscle specimens. Cleared lysates were loaded and separated on a 4–12% Bis-Tris-Protein gel (Novex NP0321BOX; ThermoFisher Scientific), followed by electroblotting. The membrane was incubated with a monoclonal antibody against human emerin as primary antibody (NCL-Emerin; Leica, used at 1:500 dilution). The SuperSignal West Femto Maximum Sensitivity Substrate (ThermoFisher Scientific) was used for antibody detection. The band corresponding to myosin heavy chain in the Coomassie-stained gel was used as loading control.

### cDNA analysis on skeletal muscle specimen

Total RNA was isolated from fresh-frozen skeletal muscle specimens using the RNeasy Fibrous Tissue Mini Kit (Qiagen, Venlo, Netherlands). RNA was reverse transcribed with the QuantiTect reverse transcription kit (Qiagen), and cDNA was analyzed by PCR and Sanger sequencing. The forward and reverse primers were designed to hybridize to different exons that were separated by large introns to generate a specific PCR product (forward primer located in exon 1 and reverse primer located in exon 3, *EMD* NM_000117.3). The β-actin gene (NM_001101.3) was used as an internal control. Primer sequences and PCR conditions are available upon request.

## Results

### Pedigree consistent with X-linked inheritance of DCM

**Fig. 1:**
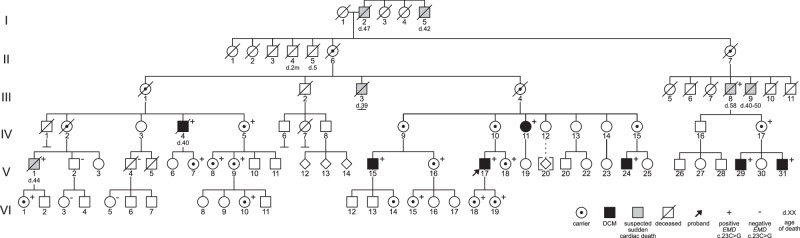
Pedigree showing the *EMD* variant c.23C>G co-segregating with DCM in an X-linked manner, and suspected sudden cardiac death in male family members. The proband is designated with an arrow. Individuals diagnosed with DCM are denoted by black-filled symbols and males deceased due to suspected sudden cardiac death are denoted by gray-filled symbols. Genetic test results are shown by “+”, indicating hemizygosity or heterozygosity for the *EMD* variant, or “−”, indicating that the *EMD* variant was not detected. In addition to “+” indicating a positive carrier test, female carriers are designated with a bull’s eye to show obligate carriers who did not undergo genetic testing.

The study included 25 living members (six male and 19 female) of a Swedish family with a history of suspected X-linked DCM (Fig. 1). Additionally, the medical records of five deceased male family members were evaluated. In total, six male family members (five living, one deceased) met the diagnostic criteria for DCM. Their clinical phenotypes are described in Table 1 and Supplementary Table 1.

**Table 1:** Clinical features of affected males hemizygous for the *EMD* variant c.23C>G.

Pedigree number	Age at diagnosed cardiac disease (years)	Age at diagnosed DCM (years)	Age at last cardiac evaluation (years)	CMRI LVEDV_index_ (ml/m^2^)	TTE LVEDD (mm)	TTE EF (%)	Left atrial dimensions	TTE findings	ECG findings	Pacemaker/ICD	Heart transplant	Histopathologic findings	Neuromuscular symptoms	CK (µkat/L)	Emerin level in myocardium	Emerin level in skeletal muscle
V:15	50	50	54 (heart transplant)		64	19	LA area 37 cm^2^	BAD, BVD	AFl, AVBI–III, LBBB, nsVT, PVCs	CRT-P, ICD	Yes	Myocyte hypertrophy and interstitial fibrosis in explanted heart, no signs of myopathy in skeletal muscle	None	4.1	Reduced	Reduced
V:17^a^	32 (AVBIII)	38	39 (heart transplant)		69	15	LA area 31 cm^2^	BAD, BVD	AF, AFl, AVBIII, nsVT, PVCs, SVT	DDD-R, ICD	Yes	Myocyte hypertrophy and interstitial fibrosis in explanted heart	None	2.4	Reduced	
V:24	29 (nsVT, PVCs)	38	38	177 (66–101)^b^	59	35	LA volume 23 ml/m^2^ (LA area 17.7 cm^2^)	LAD, LVD, decreased left ventricular motility, apical akinesia	ITW, LAH, nsVT, PVCs, SB	ICD	No	No signs of myopathy in skeletal muscle	None	1.8		Reduced
IV:4	37 (LBBB, PVCs)	39	39 (heart transplant)			13		LAD, LVD	AF/AFl, AVBI–III, LBBB, nsVT, PVCs		Yes		None			
V:29	41	41	47	187 (64–99)^b^	67	25–35	LA volume 37 ml/m^2^ (LA area 24.7 cm^2^)	LAD, LVD, decreased left ventricular motility	AVBI, ITW, paroxysmal LBBB, nsVT, PVCs, VF, VT	DDD-ADI, ICD	No	Slight myocyte hypertrophy, slight interstitial fibrosis, increased endocardial thickness, and subendocardial fibrosis in right ventricular myocardial biopsy	None	1.2	Absent	
V:31	36	36	36	180 (66–101)^b^	65	20–30	LA volume 47 ml/m^2^	LAD, LVD	ITW, PVCs	ICD	No		None	2.6		

Information was collected from the last clinical control at the time of inclusion in the study. For the three heart-transplanted males, information on the cardiac features was collected from the last clinical control prior to heart transplantation.

*AF* atrial fibrillation, *AFl* atrial flutter, *AVB* atrioventricular block, *BAD* biatrial dilatation, *BVD* biventricular dilatation, *CK* creatine kinase, *CMRI* cardiac magnetic resonance imaging, *CRT-P* cardiac resynchronization therapy-pacemaker, *DCM* dilated cardiomyopathy, *DDD-R* dual-chamber rate-modulated pacemaker, *ECG* electrocardiography, *EF* ejection fraction, *ICD* implantable cardioverter defibrillator, *ITW* inverted T waves, *LA* left atrium, *LAD* left atrial dilatation, *LAH* left anterior hemiblock, *LBBB* left bundle branch block, *LVD* left ventricular dilatation, *LVEDD* left ventricular end-diastolic diameter, *LVEDV* left ventricular end-diastolic volume, *nsVT* non-sustained ventricular tachycardia, *PVC* premature ventricular contraction, *SB* sinus bradycardia, *SVT* supraventricular tachycardia, *TTE* transthoracic echocardiogram, *VF* ventricular fibrillation, *VT* ventricular tachycardia.

^a^Proband.

^b^Normal reference values [49, 50].

The six-generation pedigree was consistent with the X-linked inheritance of DCM (Fig. 1), with the six affected males being related through non-symptomatic females and one male who died of suspected SCD at the age of 58. Family history revealed an additional five males who died unexpectedly between the ages 39 and 50.

### Missense variant in the *EMD* gene identified by genetic screening

**Fig. 2:**
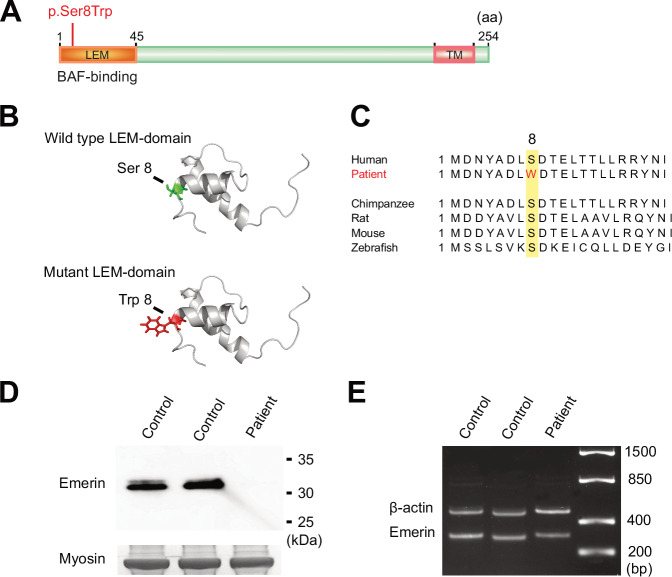
Characteristics of the *EMD* variant c.23C>G, p.Ser8Trp and effect on *EMD* expression. **A** Schematic diagram of emerin (P50402) showing the location of the N-terminal LEM domain and the C-terminal transmembrane (TM) domain. The location of the missense variant identified in the family, c.23C>G, p.Ser8Trp, is marked in red. **B** Illustration of the stereo view of the emerin LEM domain. The exchange from serine (Ser), marked in green in the wild type, to tryptophan (Trp), marked in red in the mutant, is shown. **C** Illustration showing the evolutionary conservation of the amino acid sequence in emerin. The position of the exchange, 8, is highlighted in yellow and the exchange from serine (S) to tryptophan (W) in the patient is marked in red. **D** Western blot analysis on skeletal muscle lysates from individual V:15 (patient) and two age-matched controls showing that emerin is almost completely absent and that no aberrantly spliced protein was detected in the patient compared to the control samples. The band corresponding to myosin heavy chain in the Coomassie-stained gel was used as loading control. **E** *EMD* expression was investigated via reverse transcriptase polymerase chain reaction followed by PCR and Sanger on RNA extracted from skeletal muscle tissue from individual V:15 (patient), showing transcripts at near normal levels compared to two age-matched controls samples and no aberrant spliced transcripts were detected. The β-actin gene was used as a loading control.

Genetic screening of two affected males, V:17 and V:29, using broad exome-based gene panels for DCM identified a missense variant in the *EMD* gene located on the X-chromosome, c. 23C>G, p.Ser8Trp (NM_000117.3). The *EMD* c.23C>G variant is predicted to result in substitution of serine for tryptophan in position 8 in the LEM domain of the emerin protein (Fig. 2A, B). The position is conserved in mammals (Fig. 2C) and there is a large physiochemical difference between serine and tryptophan (Grantham distance 177). Computational prediction tools provide conflicting interpretations of pathogenicity. It was absent in the Genome Aggregation Database version 4.1.0 as of 2023. At the time of genetic screening, the *EMD* c.23C>G variant had not been previously reported but has since been submitted to ClinVar on one other occasion (ClinVar accession number VCV000222597.2).

### Co-segregation of the *EMD* variant with DCM

Targeted testing of the remaining four affected males, V:15, V:24, IV:4, and V:31, confirmed that all six were hemizygous for the *EMD* variant c.23C>G. Additionally, two male family members not harboring the *EMD* variant, V:2 and V:4, were confirmed to be unaffected by the age of 63 and 66 years, respectively. A segregation analysis was conducted for these male family members. Those deceased due to suspected SCD were not included since a diagnosis of underlying DCM could not be confirmed by a review of their medical records.

The LOD score was estimated at 3.9, with values above 3.0 considered evidence of linkage between the *EMD* locus and DCM. Calculations of the FLB yielded odds of 4617:1 in favor of causality. Since the exact penetrance, phenocopy rate and allele frequency were unknown, the analysis was repeated systematically with a range of more conservative values and constantly yielded odds above 2500:1 in favor of causality. The occurrence of other potential disease-causing variants in the *EMD* gene or linkage interval was excluded by manual examination of whole genome sequencing data in V:29.

### Emerin deficiency in cardiac and skeletal muscle

**Fig. 3:**
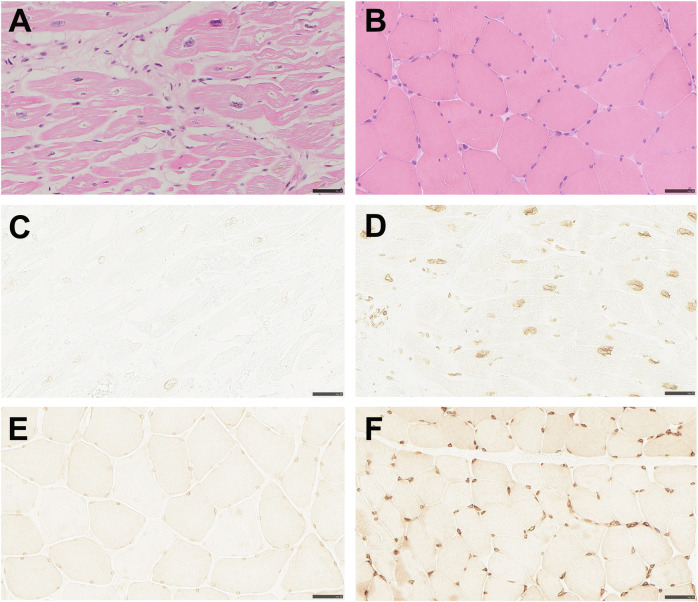
Histopathology and emerin immunostaining in samples collected from individual V:15 showing myopathy in cardiac muscle but not skeletal muscle while emerin immunostaining is markedly reduced in both tissues. Bars correspond to 50 µm. **A** The myocardium of the explanted heart shows marked cardiomyocyte hypertrophy and increased interstitial connective tissue (hematoxylin and eosin staining). **B** The skeletal muscle specimen collected at age 61 shows no signs of myopathy (hematoxylin and eosin staining). **C** Heart explant showing markedly reduced emerin immunostaining of nuclear membranes. **D** Heart specimen from control with normal immunostaining of nuclei. **E** Skeletal muscle specimen showing markedly reduced emerin immunostaining of nuclear membranes. **F** Skeletal muscle specimen from control with normal immunostaining of nuclei.

Myocardial samples were available from three affected males (two explanted hearts and one endomyocardial biopsy), and deltoid muscle samples from two affected males. Histopathological examination of the myocardial specimens showed cardiomyocyte hypertrophy and interstitial fibrosis (Table 1; Fig. 3A) consistent with DCM. Immunohistochemical staining for emerin was conducted on the myocardial specimens from the two explanted hearts, showing greatly reduced levels of emerin in nuclear envelopes of cardiomyocytes from the affected males, although not complete absence (Table 1; Fig. 3C), when compared with a control sample (Fig. 3D). In the specimen from the endomyocardial biopsy, immunostaining showed complete absence of emerin.

Greatly reduced emerin levels in the nuclear envelope of myocytes were also seen in the two available deltoid muscle samples (Table 1; Fig. 3E), when compared with a control (Fig. 3F). To quantify the level of residual emerin, Western blot on skeletal muscle tissue from one affected male was conducted, resulting in levels of full-length emerin too low to quantify and only just visible by overexposure (Fig. 2D). cDNA analysis performed on the same sample confirmed that the *EMD* gene was transcribed and that mRNA isolated from skeletal muscle included the missense variant and showed no signs of aberrant splicing (Fig. 2E).

### Muscular dystrophy absent in all affected males

There was no history of neuromuscular symptoms in any of the six affected males. The five living affected males underwent neuromuscular examination without findings of joint or skeletal muscle symptoms associated with EDMD1, at ages 61, 47, 37, 47, and 37. Serum-creatine kinase levels were normal. Deltoid muscle samples from the two affected males biopsied at ages 61 and 37 showed no evidence of myopathy on histopathological examination (Table 1; Fig. 3B). None of them reported limitations regarding strength or flexibility despite active lifestyles.

### Cardiac phenotypes in affected males

**Fig. 4:**
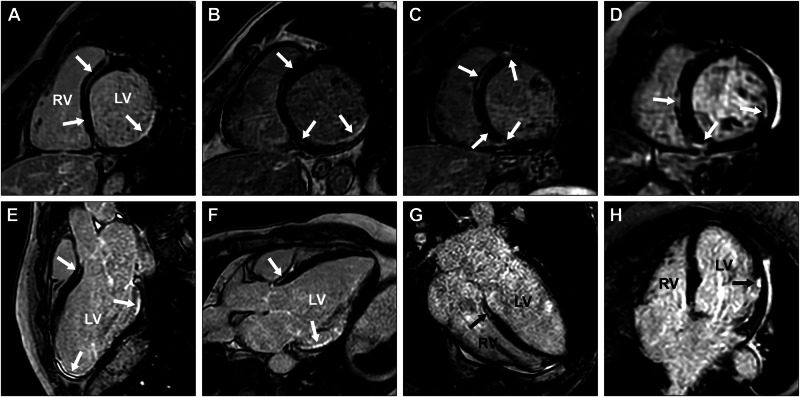
CMRI showing both ischemic and non-ischemic LGE patterns. CMRI revealed partly similar delayed enhancement patterns in the short- (**A**–**D**) and long-axis projections (**E**–**H**) of individuals V:24 (**A**, **E**), V:29 (**B**, **F**), V:31 (**C**, **G**), and IV:11 (**D**, **H**). A detailed description of all LGE findings (indicated by white and black arrows) can be found in Supplementary Table 3. The left ventricle is denoted by LV and the right ventricle by RV.

The six affected males were diagnosed with DCM between ages 36 and 50, and three of them had progressed to end-stage heart failure and undergone heart transplantation. Cardiac conduction defects and ventricular arrhythmias were frequent, requiring implantable cardioverter defibrillator (ICD) in all five living affected males. In three, premature ventricular contractions (PVCs), non-sustained ventricular tachycardia (nsVT), or complete atrioventricular block were documented while ventricular dimensions were still normal, 2 to 9 years before they met the criteria for DCM. One of the affected males experienced malignant ventricular arrhythmia including several episodes of sustained ventricular tachycardia and one episode of ventricular fibrillation aborted by ICD shock. Left ventricular fibrosis was detected in the five living affected males examined, either by histopathological examination or visualized as late gadolinium enhancement (LGE) by CMRI. CMRI revealed both ischemic and non-ischemic distribution of LGE. Detailed information concerning CMRI findings in affected males is presented in Fig. 4A–C, E–G, and Supplementary Table 3.

### Early-stage disease progression documented in one affected male

For one of the affected males, V:24, cardiac investigations were initiated due to the detection of ECG abnormalities at a routine medical check-up, which permitted monitoring of the early stages of disease progression for 9 years. The first 24-h ECG recording, at 29 years of age, showed up to 6880 (5.5%) PVCs as well as several short episodes of nsVT. At the time, left ventricular dimensions were normal and systolic function only slightly decreased. However, subendocardial LGE was already present in the basal inferior and inferolateral segments, as well as subendocardial to transmural LGE in all apical segments including the apex. Thus, a purely ischemic LGE pattern was initially present. It was not until the age of 37 that he fulfilled the diagnostic criteria for DCM. His most recent CMRI, at age 38, showed severe left ventricular dilatation and systolic dysfunction with decreased ventricular motility. Furthermore, widespread LGE, including a non-ischemic LGE pattern was now visible (Fig. 4A, E; Supplementary Table 3).

### Cardiac phenotypes in female *EMD* variant carriers

Initially, there were no reported cases of DCM in female family members. Subsequent carrier testing in the family identified 17 females heterozygous for the *EMD* variant c.23C>G (four obligate carriers were not confirmed by genetic analysis) (Fig. 1). All female carriers were referred for cardiac monitoring. Thirteen females underwent combined cardiac ultrasonography and ECG, three only cardiac ultrasonography, and one only ECG. Ages ranged from 20 to 87 (median age 42).

Cardiac monitoring yielded normal results for all female carriers, except one who at age 71 presented with frequent PVCs on ECG, while left ventricular end-diastolic diameter (42 mm) and ejection fraction (60%) were still normal. Three years later, at 74 years of age, she was diagnosed with heart failure due to DCM. CMRI showed a dilated left ventricle with severely reduced systolic function, as well as widespread LGE (Fig. 4D, H; Supplementary Table 3). ECG showed frequent PVCs and nsVT and she received an ICD. She had no ischemic heart disease at coronary angiography and no neuromuscular symptoms.

## Discussion

This study provides new support for an association between the *EMD* gene and DCM without muscular dystrophy, by presenting evidence of co-segregation in a large family, emerin deficiency in the myocardium and skeletal muscle, and normal neuromuscular examinations.

Currently, the *EMD* gene is only established as causative for DCM as a feature of EDMD1 [6, 24]. However, DCM is rarely seen in EDMD1 [11, 12] and the cardiac phenotype is generally dominated by atrial pathology due to fibroadipose tissue remodeling of the atria. This results in atrial enlargement, predominantly of the right atrium, as well as atrial arrhythmias and cardiac conduction defects in the second and third decade [11, 33], which progress to atrial stand-still in the fourth [8]. In general, ventricular dilatation is less severe and precedes ventricular arrhythmias which develop in the sixth decade [8, 11]. Importantly, even in EDMD1 cases with mild or no neuromuscular symptoms, the cardiac phenotype is usually consistent, with predominantly atrial pathology [18, 19, 27, 28, 34, 35].

Our results indicate that isolated DCM might be a separate *EMD* phenotype, as opposed to an instance of phenotypical variability. Firstly, neuromuscular symptoms were completely absent in all six males harboring the *EMD* variant c.23C>G. Detailed neuromuscular examination in adulthood excluded even subtle signs of muscular dystrophy, which was emphasized by normal skeletal muscle morphology on histopathological examination. Secondly, the progression of cardiac symptoms in the six affected males differed from that of EDMD1. The atrial pathology in the transplanted males, with predominantly left-sided dilatation and atrial fibrillation and flutter, was considered secondary to ventricular dilatation, and no early atrial manifestations were present in the three males who had not reached end-stage DCM.

Instead, the cardiac phenotype was characterized by severe arrhythmogenic DCM with onset in the fourth or fifth decade in males. In one affected male, V:24, disease progression was documented 8 years before DCM diagnosis. He initially showed an ischemic LGE pattern on CMRI and a high frequency of PVCs and nsVT on ECG, several years before dilatation of the left ventricle, corresponding to non-dilated left ventricular cardiomyopathy preceding DCM development [1]. Given the ischemic pattern of LGE, early stages of disease progression could easily be misdiagnosed as ischemic cardiomyopathy, emphasizing the need for coronary angiography and for the clinician to be alert to differential diagnoses. Myocardial fibrosis was also seen in the two other affected males examined with CMRI, including septal intramural LGE which has been associated with greater arrhythmic risk than other patterns [36]. Furthermore, malignant ventricular arrhythmias were prominent in one male and the family history revealed several cases of suspected SCD.

Taken together, the cardiac phenotype in the family differed from that of classical EDMD1 and shared common features with *LMNA*-related DCM, such as cardiac conduction defects and ventricular arrhythmias preceding DCM development, which warrant consideration of early ICD implantation due to the risk of cardiac arrest. This notion is supported by results from a cohort study by Cannie et al., indicating that male *EMD* variant carriers had a risk of malignant ventricular arrhythmias and heart failure similar to that of *LMNA*-variant carriers [13]. The similarities with *LMNA*-related DCM are highlighted by the shared LGE pattern, with both ischemic [37] and non-ischemic distribution [1], which can be helpful in recognizing this phenotype. These similarities might be explained by shared functions between emerin and lamin A/C, encoded by the *LMNA* gene, which are binding partners in the nuclear envelope [38]. In addition, the cardiac features described here also overlap with other genetic arrhythmogenic cardiomyopathies with predominant left ventricular involvement, such as *FLNC*-related cases. *EMD* may therefore be considered along with *LMNA*, *FLNC*, and other similar genes for genetic screening in men presenting with the described pattern.

In our study, the *EMD* c.23C>G variant was shown to co-segregate with X-linked DCM based on an estimated LOD score above 3.0. To account for the risk of phenocopies, the calculation of FLB [29] was also performed, yielding odds above 2500:1 in favor of causality. There are no generally accepted thresholds for FLB, although for single families 32:1 has been suggested as strong evidence for co-segregation [39] and previously applied to this method [30], which illustrates the strength of our evidence of co-segregation. Additionally, the *EMD* c.23C>T variant has been reported in three males from a cohort of EDMD1 with cardiac disease, further supporting causality although detailed phenotype information was not provided [13]. To our knowledge, the only previous evidence on co-segregation between *EMD* and isolated DCM was of a founder-variant (c.77T > C) on Tenerife Island, where hemizygous males from 20 separate families displayed the same cardiac-specific phenotype of severe arrhythmogenic DCM as in our study [21, 22].

By immunohistochemical staining for emerin, we demonstrated greatly reduced emerin levels in both myocardium and skeletal muscle, strongly indicating a defect in the *EMD* gene with an effect on the encoded protein in both tissues. Immunohistochemical detection of emerin deficiency in skeletal muscle is considered diagnostic for EDMD1 [7, 40] and presents a clinically available method of investigating the functional effect of new *EMD* variants.

While our study links *EMD* to isolated DCM, we expect *EMD* variants to have a limited contribution to the overall disease burden. This was demonstrated in a recent large case-control study showing no difference in the distribution of *EMD* variants in DCM patients and controls [23], which might be due to the rarity of *EMD* variants [41], and possibly accentuated by the autosomal dominant model of the study. Hence, further strong case-level data are necessary to determine the role of *EMD* in DCM.

Of the 17 female carriers included in our study, one developed DCM after 70 years of age, with a similar presentation as her affected male relatives. Literature data on the penetrance of cardiac disease in females in the context of EDMD1 are limited. In one study of 30 *EMD* variant carriers, five had cardiac symptoms including atrioventricular block or atrial fibrillation [42]. In another study, nine of 21 female *EMD* variant carriers developed cardiac disease at a median age of 58.6 [13]. Taken together, the data suggest an increased risk of cardiac disease in female carriers, and cardiac monitoring may therefore be indicated.

A limitation of this study was that the participants were characterized in the context of clinical care, and clinical examination, imaging, and immunohistochemical data were not consistently obtained across all patients. There are also limitations adhered to studying a single family, and to validate our findings they need to be confirmed in additional families.

A further limitation is that our study does not provide an explanation for why the *EMD* variant c.23C>G results in a cardiac-specific phenotype, despite equal emerin deficiency in cardiac and skeletal muscle. Given the greatly reduced, although not completely absent, levels of emerin in both tissues, small amounts of residual full-length emerin in the inner nuclear membrane of myocytes might be sufficient to rescue skeletal but not cardiac muscle function. However, studies of phenotype correlations with partial emerin deficiency are scarce and do not lend clear support for a cardiac-specific phenotype [43].

*EMD* null variants, resulting in complete emerin deficiency, are the predominant cause of EDMD1 [7, 40]. Further research is needed to understand by which mechanism the c.23C>G missense variant in *EMD* results in emerin deficiency, considering that the variant was not shown to alter gene transcription or splicing. To investigate if there are specific locations and sequence impacts in the *EMD* gene that may lead to isolated DCM, we performed a literature search with the aim to identify all *EMD* variants reported in association with predominant cardiac phenotypes (Supplementary Fig. 3; Supplementary Table 4). This literature review could only identify two *EMD* variants associated with isolated DCM: the previously mentioned variant c.77T > C described in multiple families by Cuenca et al. [21], and c.23C>T described in an individual case by Ishikawa et al. [17]. In addition, we are aware of one abstract describing a family with isolated DCM due to the variant c.65C > T [44]. These three variants and the c.23C>G variant in our study have all in common that they are located in the LEM domain of emerin, a conserved region between LEM-domain proteins with functions of importance for chromatin organization, regulation of gene expression, and nuclear assembly at mitosis [45, 46]. Additionally, LEM-domain alterations in proteins encoded by *LEMD2* and *TMPO* have recently been associated with arrhythmogenic cardiomyopathy with DCM combined with juvenile cataract [47], and isolated DCM [48], respectively. Taken together, the limited evidence thus far suggests that the LEM domain might be of interest for further studies to elucidate the molecular basis for the suggested association between the *EMD* gene and isolated DCM.

## Conclusion

The c.23C>G missense variant in *EMD* is associated with severe arrhythmogenic DCM without muscular dystrophy, thereby providing new evidence that *EMD* is a causative gene for isolated DCM, as a separate phenotype from EDMD1. Hence, *EMD* may be relevant to include in genetic screening for non-syndromic DCM or arrhythmogenic cardiomyopathy. Furthermore, the phenotypic similarities with *LMNA*-related DCM might warrant early interventions to prevent SCD due to malignant ventricular arrhythmias.

## Supplementary information

Supplementary Material

## Data Availability

The data supporting the findings of this study are available from the corresponding author upon reasonable request.
